# Effect of DU meridian acupuncture and temporal three-needle on vascular protection mechanisms in patients with acute cerebral infarction: a randomized controlled evaluator-blinded clinical trial

**DOI:** 10.3389/fneur.2025.1528340

**Published:** 2025-04-07

**Authors:** Yawen Sun, Qiqi Zhong, Yingci Hou, Xin Liu, Mingyue Yan, Luolin Zhou, Shiyi Liu, Senkai Hong, Jun He

**Affiliations:** ^1^First Clinical Medical College of Guangzhou University of Chinese Medicine, Guangzhou, China; ^2^The First Affiliated Hospital of Guangzhou University of Chinese Medicine, Guangzhou, China

**Keywords:** acupuncture, Du meridian, acute cerebral infarction, vascular protection, protocol

## Abstract

**Background:**

The incidence of acute ischemic stroke has been rising steadily in China and globally, with its high mortality and disability rates significantly affecting quality of life. As an important adjunct therapy, acupuncture has been widely implemented in stroke management. Emerging studies have investigated the effects of DU meridian and Chong Mai acupuncture on ischemic hemiparesis; however, the vascular protective mechanisms of electroacupuncture therapy targeting these meridians require further elucidation in stroke rehabilitation research.

**Objective:**

This prospective cohort study aims to investigate the clinical efficacy and limitations of DU meridian acupuncture combined with Temporal Triple Needling (Sanjian) therapy in stroke rehabilitation. The intervention’s therapeutic potential is evaluated through its modulatory effects on CD14+/CD14- monocyte subpopulations within peripheral blood mononuclear cells (PBMCs), with particular focus on angiogenesis-mediated vascular protection mechanisms.

**Methods:**

Sixty-six patients with acute ischemic stroke will be randomly assigned 1:1 to control (conventional basal therapy, *n* = 33) and acupuncture (conventional standard stroke care + acupuncture, *n* = 33) groups for 10 days of intervention. The primary outcome is NIHSS score. The secondary outcomes: BMI and mRS scores, the level of TNF-A and IL-1B in serum, vascular endothelial growth factor (VEGF), Endocan ES, CD14+ and CD14- levels of peripheral blood mononuclear cells.

**Discussion:**

The aim of this study is to investigate whether electroacupuncture targeting the DU meridian combined with Temporal Triple Needling (Sanjian) improves cerebrovascular endothelial function in acute ischemic stroke patients, thereby reducing the NIHSS score and preventing further disease progression. This study also aims to contribute positively to the development of relevant clinical treatment protocols and to facilitate further research into the underlying mechanisms of these effects.

**Clinical trial registration:**

International Traditional Medicine Clinical Trial Registry ITMCTR2024000508.

## Introduction

Stroke is currently recognized as the second leading cause of disability and mortality worldwide (1), with acute ischemic stroke accounting for approximately 85% of cases (2). In China, the incidence of stroke has been increasing annually over the past 30 years, making it the leading cause of death in the country (3). Stroke is characterized by high incidence rates, high recurrence rates, high disability rates, and high case fatality rates (4). Acute ischemic stroke (AIS) refers to a group of syndromes caused by various factors leading to impaired blood supply to brain tissue (5). This impairment triggers a series of cascade reactions, resulting in ischemic and hypoxic necrosis in the brain, as well as neurological deficits in clinical practice (6). The current preferred treatment methods include intravenous thrombolysis and intravascular intervention therapy (7). However, treatment for AIS is often delayed due to factors such as low public awareness, a narrow thrombolysis time window, and strict eligibility criteria for thrombolysis. Only 21.5% of AIS patients in China arrive at the emergency department within 3 h of onset, and 12.6% are eligible for thrombolysis treatment, with fewer than 3% actually receiving it. The average interval between patient arrival at the emergency department and the administration of thrombolytic drugs is 116 min, significantly longer than in developed countries (8). Very few patients in China receive timely and effective thrombolytic therapy within the designated time window; therefore, it is essential for these patients to seek alternative treatments. Acute cerebral infarction is classified as a type of “stroke” in traditional Chinese medicine. Acupuncture, an important adjunctive treatment during the acute phase of cerebral infarction (9), has been shown to promote collateral circulation and improve brain circulation and metabolism to some extent (10). Currently, some scholars recommend initiating acupuncture treatment as soon as the patient’s vital signs stabilize (11). Therefore, in the early stages of acute ischemic stroke, when thrombolysis is no longer feasible and medical conditions are underdeveloped, acupuncture may offer timely intervention to mitigate the extent of cerebral infarction, reduce sequelae, and decrease the likelihood of functional disability, thereby conserving social resources (12).

## Materials and methods

### Participants

Emergency inpatients admitted to the First Affiliated Hospital of Guangzhou University of Chinese Medicine, who meet the diagnostic criteria for acute cerebral infarction as outlined in the “Guidelines for the Prevention and Treatment of Stroke in China” (2021 edition) (13), and who provide informed consent, will be included.

### Study design

**Figure 1:**
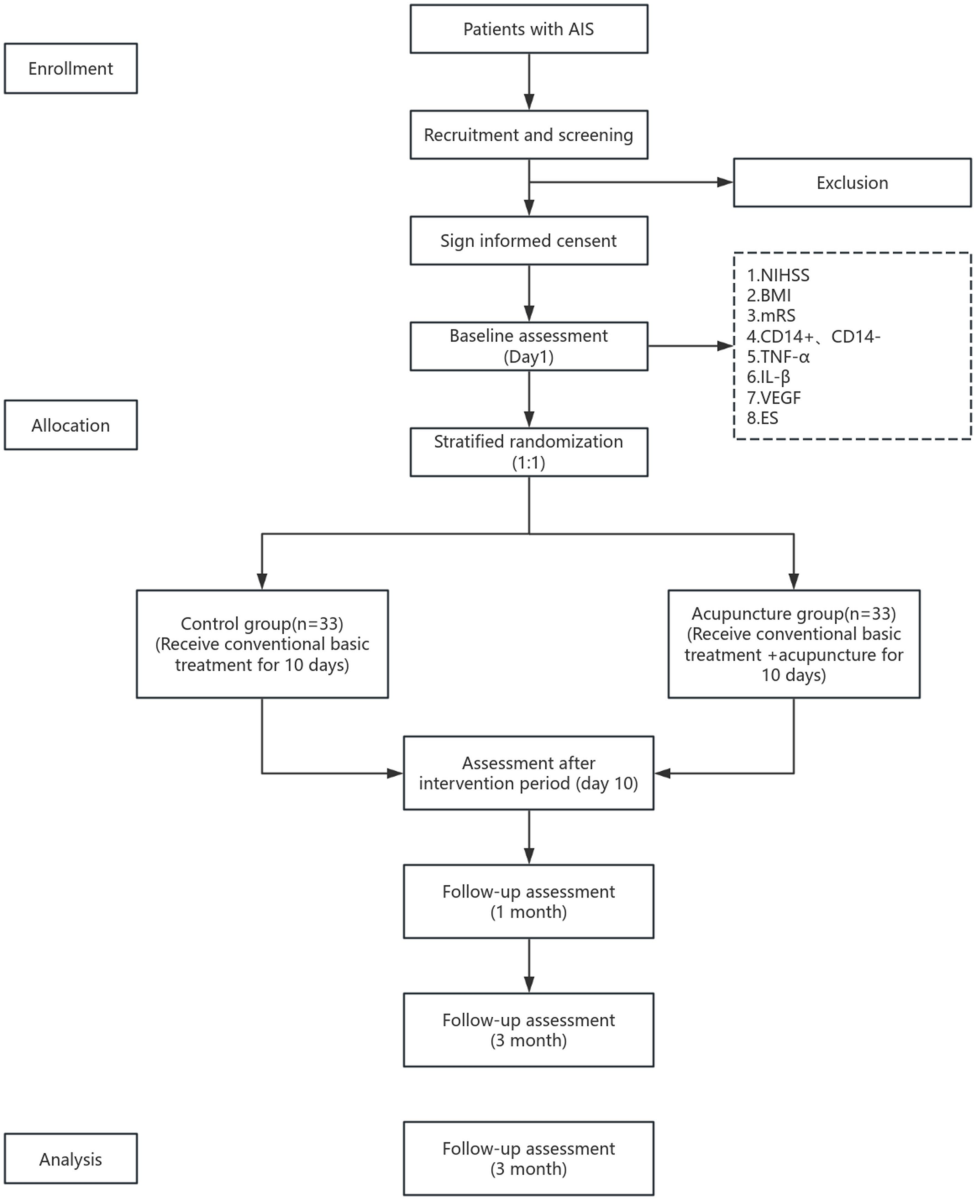
Flow chat of the study.

This is a randomized controlled trial with evaluator blinding to be conducted at the First Affiliated Hospital of Guangzhou University of Chinese Medicine. A total of 66 emergency inpatients will participate in this study. The experimental flowchart is shown in Figure 1. This study has received approval from the Ethics Committee of the First Affiliated Hospital of Guangzhou University of Traditional Chinese Medicine (Ethics Acceptance Number: JY2024-116).

### Diagnostic criteria

(1) Diagnostic criteria: The diagnostic criteria for acute cerebral infarction will refer to the “Guidelines for the Prevention and Treatment of Stroke in China” (2021 edition) formulated by the Neurology Branch of the Chinese Medical Association (9).
(2) Traditional Chinese Medicine (TCM) Diagnosis criteria: The diagnostic criteria for disease classifications are based on the “Diagnosis and Efficacy Evaluation Criteria for Stroke (Trial)” developed by the National Research Collaboration Group on Traditional Chinese Medicine Brain Disease Emergencies under the State Administration of Traditional Chinese Medicine.

### Inclusion criteria

(1) Meets the diagnostic criteria for acute cerebral infarction as outlined in the “Guidelines for the Prevention and Treatment of Stroke in China” (2021 edition), and diagnosed through head CT or MRI examination;
(2) Meets the diagnostic criteria for stroke as defined by Traditional Chinese Medicine (TCM);
(3) Individuals experiencing their first stroke within 7 days of onset and are aged between 35 and 75 years;
(4) NIHSS score ≥ 3 and ≤ 20, with no physical disability related to the study prior to disease onset;
(5) Clear consciousness, defined as GCS score ≥ 13 points;
(6) Individuals who voluntarily participate in this study and provide informed consent form.

### Exclusion criteria

Patients who meet any of the following criteria will be excluded from the study:

(1) Transient ischemic attacks, subarachnoid hemorrhage, cerebral hemorrhage, or other similar conditions;
(2) Patients requiring arterial or venous thrombolysis or thrombectomy based on their condition;
(3) Individuals suffering from immune-related diseases, such as acute or chronic infections, tumors, and blood disorders, or who have severe organ dysfunction;
(4) Individuals with severe cardiovascular dysfunction, upper gastrointestinal bleeding, intolerance to electroacupuncture, or those with pacemakers or stents;
(5) Pregnant, pre-pregnant, or lactating individuals;
(6) Family members or patients who exhibit poor initiative or lack cooperation;
(7) Participants who have participated in other clinical trials within 1 month prior to this study.

### Termination criteria

Patients who meet any of the following criteria will be excluded from the study:

(1) Individuals who experience disease progression during the study that affects vital signs and requires transfer to the intensive care unit (ICU);
(3) Individuals who experience serious adverse reactions or other events that impede recovery during the study;
(4) Patients or family members who are unwilling to continue the clinical trial and wishing to withdraw for conciseness;
(5) Individuals who exhibit intolerance to electroacupuncture or significant toxic side effects from medication during the treatment process;
(6) Clinicians determine that other circumstances warrant termination of the study.

### Sample size

Based on a review of previous research literature and the sample size calculation formula Health Statistics (14), equivalence tests were used to estimate the sample size. After 2 weeks of treatment, the NIHSS score for 63 patients in the observation group (conventional drug + acupuncture) was 7.53 ± 4.04, while the score for 63 patients in the control group (conventional drug only) was 12.61 ± 5.86. To calculate the sample size using an independent samples t-test, we need: effect size (Cohen’s d), significance level (*α*), power (1-*β*), and degrees of freedom (df). Cohen’s d is used to calculate the effect size, and the formula for Cohen’s d is: $d=\frac{M1-M2}{{SD}_{\mathrm{pooled}}}$ . For the observation group (conventional medication + electroacupuncture treatment): mean M1 = 7.53, standard deviation SD1 = 4.04, and for the control group (conventional medication): mean M2 = 12.61, standard deviation SD2 = 5.86. The pooled standard deviation (SDpooled) is known to be 5.62. Substituting into the formula, we obtain d ≈ −0.9, which represents a large effect size. When calculating the sample size using power analysis, the effect size d = 0.9, the significance level is typically set at *α* = 0.05, and the power 1 − *β* = 0.9. The formula for the independent samples t-test is: $n=\frac{2{\left(\upsilon_{\alpha }+\upsilon_{\beta }\right)}^{2}\sigma^{2}}{\delta^{2}}$ . With α = 0.05, β = 0.1, and a bilateral test, u₀.₀₅ = 1.96, u₀.₁ = 1.28, it was assumed that the control and treatment groups had equal therapeutic effects, with a combined $s=\sqrt{\frac{\left(n_{1}-1\right)s_{1}^{2}+\left(n_{2}-1\right)s_{2}^{2}+\frac{n_{1}n_{2}}{n_{1}+n_{2}}{\left({\bar{x}}_{1}-{\bar{x}}_{2}\right)}^{2}}{n_{1}+n_{2}-1}}$ standard deviation of σ=5.62 . The mean difference between the two groups was substituted into the formula $n=\frac{2{\left(1.96+1.28\right)}^{2}\times 5.62^{2}}{{\left(12.61-7.53\right)}^{2}}=25.7\approx 26$ , resulting in a sample size of 26 patients for each group, resulting in a total of 52 patients across both groups (15). Based on the Guiding Principles for Clinical Research of New Chinese Medicines, and accounting for a 20% dropout and follow-up rate, a total of 66 patients should be included.:33 patients in the control group (basic treatment) and 33 patients in the treatment group (basic treatment + acupuncture) (16).

### Random allocation

A total of 66 patients were enrolled using simple randomization with a random number table and randomly assigned to the treatment group and the control group. The group assignments and treatment protocols were placed in opaque envelopes and kept by the attending physicians and acupuncture therapists, who administered the treatment according to the instructions in the envelopes.

### Blinding

Due to the unique nature of the experimental treatment methods, implementing blinding posed challenges. In this study, grouping information will be concealed only from laboratory personnel, while clinical scales and statistical data will be collected and analyzed by dedicated staff. To strengthen methodological rigor, we have implemented full blinding for both outcome assessors (including NIHSS-certified raters) and statisticians throughout the trial process. This enhanced blinding strategy effectively prevents measurement bias that might arise from investigator anticipation or participant expectations, thereby improving the validity and objectivity of outcome evaluations.

### Interventions

(1) Control group (conventional basic treatment): 33 patients

Standardized basic treatment will follow the “Chinese Guidelines for Stroke Prevention and Control” (2021 edition): ① Antiplatelet therapy: the use of antiplatelet aggregation drugs, including oral enteric-coated aspirin tablets (Bayer Medical and Health Co., Ltd., National Medical Approval No. J20171021) or oral clopidogrel bisulfate tablets (Lepu Pharmaceutical Co., Ltd., National Pharmaceutical Approval Letter H20123116); ② Lipid-regulating and plaque-stabilizing agents: oral atorvastatin calcium tablets (Beijing Jialin Pharmaceutical Co., Ltd., National Medical Approval No. H20093819). These medications will be administered continuously for 10 days. If patients have other underlying conditions, blood pressure, blood glucose levels, and water-electrolyte balance will be regulated based on individual circumstances.

(2) Acupuncture group (conventional basic therapy + acupuncture): 33 patients

① Basic treatment: Identical to that of the control group
② Acupuncture treatment

**Figure 2:**
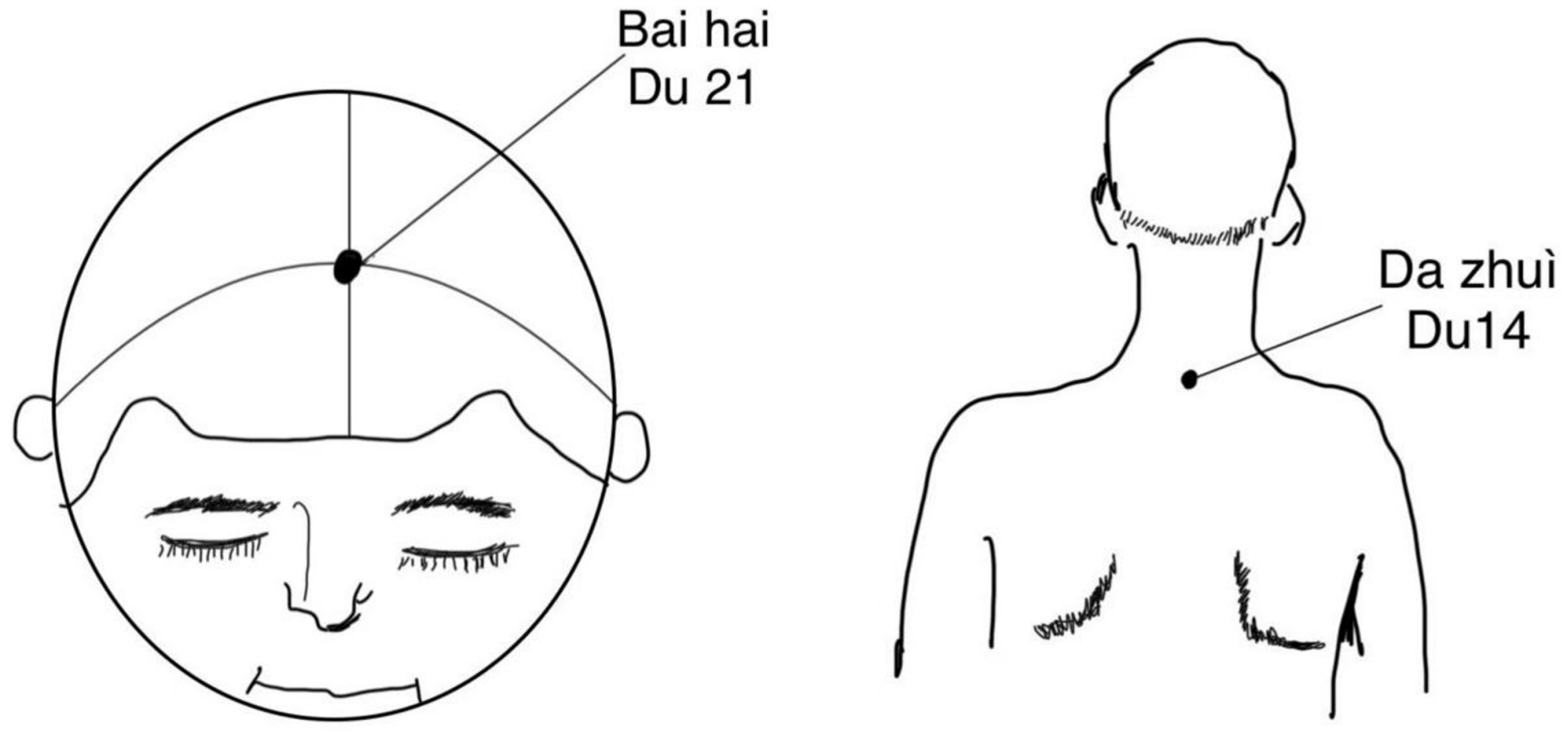
Acupoints of Baihui (Du 21) and Dazhui (Du 14).

**Figure 3:**
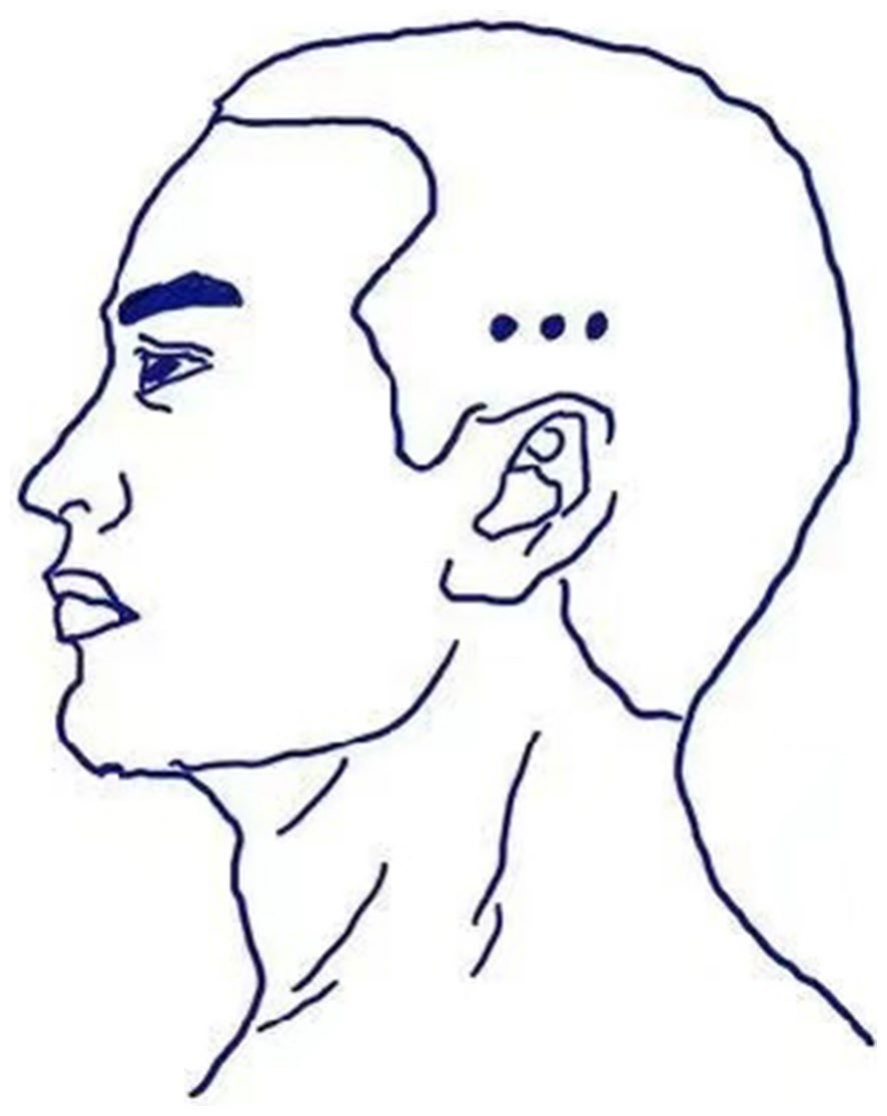
Acupoints of temporal three-needle.

- Point Selection and Positioning: The DU meridian Baihui (DU20), Dazhui (DU14; Figure 2), and temporal three-needle are selected based on acupuncture and moxibustion (17) and Jin’s Three Needle Therapy (18). Baihui (DU20) acupoint positioning: Located 5 inches above the center of the head at the current hairline, or at the midpoint of the line connecting the tips of both ears. Dazhui (DU14) acupoint positioning: Located on the posterior midline, in the depression below the 7th cervical spinous process. Positioning of temporal three-needle: Temporal I needle: Located 2 inches above the hairline at the ear tip. Temporal II needle: 1 inch forward and laterally from Temporal I needle. Temporal III needle: 1 inch backward and laterally from Temporal I needle (Figure 3). Needles: Acupuncture needles, 1-inch in length, produced by Suzhou Tianxie Acupuncture Instrument Co., Ltd. (Production lot number: SuμRegistration 20,162,200,894)
- Attention: After acupuncture treatment, patients should apply pressure to the needle site to prevent subcutaneous bleeding and cleanliness. During the treatment period, patients should avoid consuming raw and cold fruits, shrimp, beef, mutton, and other similar foods.
- Course of Electroacupuncture: Needles will be retained for 30 min per session, once daily, for a continuous duration of 10 days.
- The acupuncture points of Du-14 (Dazhui), Du-20 (Baihui), and the temporal three-needle points will be connected to the positive and negative terminals of the G6805 electroacupuncture device (Qingdao Xinsheng Industrial Co., Ltd.). A dispersive-dense wave will be selected, with a frequency of 50 Hz and an intensity of 1 mA, and electroacupuncture treatment will be performed for 30 min. No manual needle manipulation will be performed during needle retention to eliminate inter-practitioner variability.

### Outcome measurement

Clinical indicators will be conducted uniformly by professional physicians or therapists. NIHSS scores will be assessed once before treatment (Day 1), during treatment (Day 5), and after treatment (Day 10). BMI and mRS scores will be assessed before treatment (Day 1) and after treatment (Day 10).

## Main results

The National Institutes of Health Stroke Scale (NIHSS) (19, 20), consists of 11 assessment items: degree of consciousness, ability to answer questions, ability to follow instructions, eye movements, visual fields, facial muscle strength, upper and lower extremity motor function, limb coordination, sensory functioning, speech, phonology, and sensory neglect. Each item is scored on a scale of 0 to 4, resulting in a total score ranging from 0 to 42, with higher scores indicating more severe neurological damage. Scores of 0–1 indicate normal or near-normal status; 2–4 indicate mild stroke; 5–15 indicate moderate stroke; 15–20 indicate moderate to severe stroke; and scores of 20 or more indicate severe stroke, with higher scores reflecting more severe conditions. To ensure the reliability and consistency of the NIHSS scoring, all assessors will undergo standardized training. The training will cover a detailed explanation of the scoring criteria, common scoring errors, and methods to avoid bias. The process will ensure that assessors fully understand the meaning of each scoring item and can accurately apply it during the actual assessment. After the training, assessors will be tested through both written exams and practical scoring to verify their proficiency. Additionally, the results of consistency tests, including the Kappa value (which reflects the strength of consistency, with values typically >0.75 indicating strong consistency), will be provided to evaluate the inter-rater reliability of the scoring.

### Secondary results

Activities of Daily Living: Modified Barthel Index (MBI); The Modified Barthel Index (MBI) (21, 22) is used to assess a patient’s functional status in activities of daily living (ADLs), with individual scores based on a series of independent behavioral measurements and a total score ranging from 0 to 100. The Barthel Index developed in 1965 by American researchers Dorothea Barthel and Florence Mahoney, is a widely used tool for assessing ADLs in rehabilitation settings, particularly in the United States. Barthel Index Assessment Criteria:

- 60 points or more: Mildly disabled, but essentially independently.
- 40 to 60 points: Moderately disabled, requiring assistance with daily activities.
- 20 to 40 points: Severely disabled, requiring significant assistance with daily activities.
- Less than 20 points: Totally disabled, completely dependence.

Assessment of Disability: The Modified Rankin Scale (mRS) (23, 24) is used to assess neurological recovery in stroke patients. mRS Scoring Criteria:

- 0 points: Asymptomatic; mild symptoms may be present, but the patient has not experienced any new functional limitations after the stroke.
- 1 point: Symptoms are present, but there is no significant disability; the patient can complete all regular responsibilities and activities.
- 2 points: Mild disability; the patient cannot perform all previously completed activities but can manage personal affairs independently.
- 3 points: Moderate disability; the patient requires some assistance but can walk independently.
- 4 points: Severe disability; the patient is unable to walk without assistance and cannot attend to their own physical needs.
- 5 points: Severe disability; the patient is bedridden and incontinent, requiring constant care and attention. Although a trained nurse is not necessary, the patient needs someone to assist them several times throughout the day and night.

### Blood serum index

In this study, enzyme-linked immunosorbent assay (ELISA) will quantify patients’ expression of inflammatory factors, including TNF-*α* and IL-1β (25), vascular endothelial growth factor (VEGF) (26), endothelial repressor ES (27),and peripheral blood mononuclear cells at pre-trial (Day 1), on-treatment (Day 5), and post-treatment (Day 10) levels. Additionally, flow cytometry will measure the CD14+ and CD14- levels of mononuclear cells in peripheral blood (28).

### Safety outcomes

The researchers will closely monitor patients during treatment, recording vital signs such as temperature, respiratory rate, pulse rate, and blood pressure. If the patient experiences any discomfort, the intervention will be stopped immediately, and appropriate treatment will be provided based on the patient’s condition. Any adverse reactions from needling, including localized redness, swelling, pain, or other events, will be documented by the operator. The study will now employ the internationally recognized Common Terminology Criteria for Adverse Events (CTCAE) to categorize and grade the severity of adverse events.

### Follow-up

Patients will be followed up at 1 and 3 months post-discharge, with assessments of daily living ability (using the MBI scale) and disability (using the mRS scale).

### Data monitoring and statistical analysis

To minimize measurement bias arising from multiple evaluators, clinical indicator assessments and laboratory tests for each patient before, during, and after treatment will be conducted by the same specialized physician whenever possible. The physician evaluating the laboratory indicators will be blinded to the group assignments and treatment. To avoid missing or incorrect data, clinical and laboratory indicator evaluations will be conducted according to preset time points and entered into the electronic database promptly.

Data management and analysis will be performed using IBM SPSS Statistics Version 26 (IBM SPSS Inc., Chicago, United States). The principles of data processing are as follows: measurement data will be expressed as mean ± standard deviation ( $\bar{x}$ ± S); if count data are normally distributed, the chi-square test will be used; if data in the two groups are normally distributed, t-test, repeated-measures analysis of variance (ANOVA), and multivariate linear regression will be used for comparative analyses; if count data are not normally distributed, the Mann–Whitney U test will be used; *p* < 0.05 will indicate statistically significant differences.

We will use the last observation carried forward (LOCF) method, which replaces missing data points with the patient’s last observed value. In order to provide a more comprehensive assessment of the treatment effects in this study, we will conduct both an intention-to-treat (ITT) analysis and a per-protocol (PP) analysis.

We will implement the Bonferroni correction during statistical analysis to rigorously control Type I error rates, thereby ensuring methodological stringency in accordance with your recommendation.

## Discussion

Acupuncture, as a therapeutic method, has a history of over 3,000 years and is widely used in China and other Asian countries for disease prevention and treatment. Acupuncture can be used to treat a variety of internal and external diseases. Currently, it is commonly used in clinical practice for stroke treatment and various pain disorders. Recent large-scale, multi-center clinical trials have shown that acupuncture has a significant therapeutic effect in treating acute ischemic stroke patients. In particular, electroacupuncture demonstrates protective effects on cerebral ischemic injury, such as improving blood circulation, reducing infarct size, and inhibiting inflammatory responses (29). In recent years, numerous animal experiments and clinical studies have revealed the molecular and biophysical interactions of acupuncture or electroacupuncture in alleviating cerebral ischemic injury. Acupuncture intervention can significantly reduce infarct size, improve cerebral blood circulation, promote regional energy metabolism, regulate lipid metabolism, resist cerebral free radical damage, inhibit cortical apoptosis, reduce excitatory amino acids, decrease neurotoxicity, alleviate cerebrovascular immune-inflammatory responses, and promote the proliferation and differentiation of neural stem cells (30). In the treatment of pain, a study has also evaluated the efficacy and safety of acupuncture point stimulation in the treatment of gastric cancer pain for the first time. This study included 11 randomized controlled trials with 768 patients, divided into acupuncture point stimulation treatment and drug control groups. The study results showed that the acupuncture point stimulation treatment group had better efficacy than the drug control group, with a greater reduction in the NRS score. No serious adverse events were reported in the acupuncture point stimulation treatment group, and it demonstrated better control of adverse reactions (31). Other clinical studies have shown that acupuncture has a positive effect in relieving myofascial pain (32). The mechanisms of action differ between stroke and pain treatments. Acupuncture alleviates neuroinflammatory responses by stimulating vagal nerve activity and cholinergic anti-inflammatory pathways, which may be a focus of future research. Electroacupuncture promotes neuroprotection and neuroregeneration, acting through various mechanisms following ischemic brain injury.

In pain management, its mechanisms are focused on peripheral and central pathways, including reducing pain transmission and enhancing the release of endogenous opioids. In the fields of cancer pain, neurological diseases, and post-stroke recovery, acupuncture demonstrates increasing therapeutic advantages, not only improving the quality of life for patients but also offering more treatment options. However, the clinical application of acupuncture still faces many challenges, including the need for more clinical studies to further validate its efficacy and mechanisms.

The aim of this study is to determine whether acupuncture could improve vascular function in patients with AIS, thereby reducing NIHSS scores and preventing further disease progression. In this randomized controlled trial, we aim to improve patients’ neurological function and daily living abilities through acupuncture. To achieve this, we selected a series of scales and outcomes to comprehensively assess the efficacy of acupuncture. To objectively assess stroke severity, we selected the NIHSS as the primary outcome, a scale that comprehensively evaluates patients’ stroke conditions. To assess the quality of daily life, we employee the Modified Barthel Index (MBI) and the Modified Rankin Scale (mRS) as secondary outcomes.

The emerging therapeutic approach for ischemic stroke focuses on vasoprotective mechanisms and promoting neovascularization. To achieve this, neovascularization must be induced through various pathways, such as promoting the expression of VEGF and EPCs, or creating favorable conditions by inhibiting the expression of ES and inflammatory factors. Downregulation of inflammatory factors and ES expression can reduce vascular injury. Additionally, the proliferation and migration of EPCs directly promote neovascularization and mobilize factors like VEGF to facilitate tissue protection and blood vessel formation. VEGF is a key regulator of angiogenesis, and acupuncture may promote VEGF release by activating the PI3K/Akt and ERK1/2 signaling pathways, thereby enhancing vascular regeneration in ischemic regions. Additionally, ES, as an angiogenesis inhibitor, may play a role in the balance of vascular remodeling regulated by acupuncture. CD14+ and CD14- monocytes are essential in inflammation and vascular repair processes, and acupuncture may modulate their functions by influencing the Toll-like receptor 4 (TLR4) and nuclear factor-κB (NF-κB) signaling pathways, exerting anti-inflammatory and vascular protective effects.

Acupuncture improves blood and oxygen supply to the ischemic hemiparesis (33), effectively protects cerebral blood vessels, and restores brain function (34). Acupuncture has been widely used to treat stroke sequelae; however, its therapeutic effects and specific vascular protection mechanisms during the acute stage of stroke remain poorly understand. Existing studies suggest that potential vascular protection mechanisms can be categorized as follows: first, the effects of pro-angiogenic factors, including the regulation of VEGF/VEGFR (35), angiopoietin and its receptor Ang/Tie, basic fibroblast growth factor (bFGF), Apelin/APJ in vascular endothelial cells, and the expression of endothelial progenitor cells (EPCs) as well as the regulation of the Wnt/*β*-catenin signaling pathway; second, the effects of inhibitory angiogenesis factors, primarily characterized by reduction of endostatin (ES) expression (36). Intervention with endothelial progenitor cells (EPCs) using “acupuncture serum” collected from acupuncture points resulted in a significant increase in the proliferation, migration, and adhesion of these EPCs (37), indicating that acupuncture promotes angiogenesis following cerebral ischemia. EPCs can be derived from the CD14+ and CD14-cell subsets of adult peripheral blood mononuclear cells. Both subsets can express marker proteins and differentiate into endothelial cells under the stimulation of growth factors, thus promoting neovascularization. However, it remains unclear whether acupuncture can effectively increase the expression levels of CD14+ and CD14- cells in peripheral blood mononuclear cells, leading to the formation of EPCs. This study aims to determine whether acupuncture can promote blood vessel regeneration by increasing the expression of these CD14+ and CD14- cells, thereby enhancing vascular protection.

During the acute phase of cerebral infarction, blood–brain barrier damage and endothelial cell degeneration occur in patients with acute ischemic stroke (AIS), accompanied by elevated levels of TNF-*α* and IL-1β (37). To elucidate the vasoprotective mechanism of DU meridian acupuncture, the levels of inflammatory factors TNF-α and IL-1βas well as vascular endothelial growth factor (VEGF), endothelial repressor (ES) expression, and CD14+ and CD14- cell levels in peripheral blood mononuclear cells (28). These serve as serological indicators for assessing improvements in vascular health.

The trial aims to assess the efficacy of DU meridian acupuncture in treating AIS and to explore whether this acupuncture method provides vascular and brain protection by effectively inhibiting inflammation, reducing apoptosis, and promoting the generation and reconstruction of collateral circulation. These findings may offer valuable insights for the development clinical treatment options for ischemic stroke and for further investigation into the underlying mechanisms of these effects. While this study focuses on comparing acupuncture with guideline-recommended baseline treatment (antiplatelet and lipid-lowering therapy), future research could benefit from exploring other treatment strategies with potential vascular protective mechanisms. Therapies such as intensified blood pressure control, endothelial-protective agents (e.g., cilostazol), or non-pharmacological interventions (e.g., aerobic exercise) could be further investigated to deepen our understanding in future studies.
